# Reply to Deng: Polygyny’s marriage squeeze has little power to explain violence

**DOI:** 10.1073/pnas.2607580123

**Published:** 2026-04-10

**Authors:** Hampton Gaddy, Rebecca Sear, Laura Fortunato

**Affiliations:** ^a^Department of Economic History, London School of Economics, London WC2A 3LZ, United Kingdom; ^b^Leverhulme Centre for Demographic Science, University of Oxford, Oxford OX1 1JD, United Kingdom; ^c^Centre for Culture and Evolution, Brunel University, London UB8 3PH, United Kingdom; ^d^Institute of Human Sciences, University of Oxford, Oxford OX2 6QS, United Kingdom; ^e^Santa Fe Institute, Santa Fe, NM 87501

In ref. 1, we analyze the demography of polygyny and male singlehood. We conclude that the claim that polygyny is a major driver of violent crime and armed conflict (e.g., refs. 2 and 3) seems unlikely in light of that demography.

In reply, Deng (4) makes several points. Regarding our analysis, they argue that the association between polygyny and bachelorhood is biased downward by confounders and could have been estimated more precisely. Regarding our conclusion, they claim that the link between polygyny and conflict “remain[s] empirically well-supported” because polygyny often delays when men marry, even though it does not necessarily produce permanent bachelors.

The direct effect of polygyny on bachelorhood depends on local demography. The effect is null when polygyny is uncommon enough that all men can still marry monogamously, and our demographic model shows that this situation is more common than often assumed. Otherwise, the effect is positive. A causally well-identified analysis will recover the total (direct plus indirect) effect in each context, but it is not clear that Deng captures these effects. Pooled estimation increases power if one is interested in the average effect of polygyny, but it ignores local variation. Moreover, at least two of the three control variables introduced appear to be “bad controls” (5). Polygyny is likely to influence sex-specific migration decisions (6). Therefore, the sex ratio mediates polygyny’s effect on bachelorhood and is not a good control. Additionally, polygyny and bachelorhood occur in dense causal webs, and both are likely to (in)directly influence education. As a result, controlling for education (alone) opens colliding paths, threatening identification (7).

The purpose of our analysis is to examine the practical importance of polygyny for bachelorhood, not its effect in a vacuum. By focusing on the unadjusted association—which is often negative—we reveal that polygyny has little predictive power over bachelorhood. In turn, polygyny has even less predictive power over conflict, at least via the mechanism of bachelorhood. Deng’s use of the wife-husband ratio to measure polygyny is valuable, but it simply reproduces the unadjusted results on which we base this argument.

Recent work concludes that the theory that polygyny gives way to monogamy by destabilizing society is poorly supported by phylogenetic analysis (8). An effect being plausible, or even real, does not mean that it is important. Returning to a point in our discussion: China has the lowest rate of permanent bachelorhood in East Asia, despite the effect of sex-selective abortion (9). As a result, it is not obvious that we should believe the argument that sex-selective abortion will drive China to domestic instability and international conflict (10). On the same grounds, we dispute Deng’s suggestion that polygyny may drive conflict because it delays when men marry. If the norm is for men in communities with high rates of polygyny to simply wait until 25 to 30 to marry (earlier than in the West), it is not obvious that they will want to join Boko Haram (3) in the meantime. Moreover, this sort of mechanism is understudied and undertheorized (11).
